# Expression of Hypoxic Marker Carbonic Anhydrase IX Predicts Poor Prognosis in Resectable Hepatocellular Carcinoma

**DOI:** 10.1371/journal.pone.0119181

**Published:** 2015-03-04

**Authors:** Wei-Ju Huang, Yung-Ming Jeng, Hong-Shiee Lai, Iok-U Fong, Fang-Yu Bonnie Sheu, Po-Lin Lai, Ray-Hwang Yuan

**Affiliations:** 1 Graduate Institute of Pathology, College of Medicine, National Taiwan University, No. 1, Jen-Ai Road, Section 1, Taipei, 10051, Taiwan; 2 Department of Nursing, Hsin-Sheng College of Medical Care and Management, No. 418, Gaoping Section, Zhongfeng Road, Longtan Township, Taoyuan County, 32544, Taiwan; 3 Department of Pathology, National Taiwan University Hospital and College of Medicine, National Taiwan University, No. 1, Jen-Ai Road, Section 1, Taipei, 10051, Taiwan; 4 Departments of Surgery, National Taiwan University Hospital and College of Medicine, National Taiwan University, No. 1, Jen-Ai Road, Section 1, Taipei, 10051, Taiwan; 5 Department of Biomedical Science, University of Illinois College of Medicine, 1601 Parkview Ave, Rockford, IL, 61107, United States of America; 6 Department of Integrated Diagnostics and Therapeutics, National Taiwan University Hospital, No. 7, Chung-Shan South Road, Taipei, 10051, Taiwan; National Yang-Ming University, TAIWAN

## Abstract

Carbonic anhydrase IX (CA-IX), a hypoxia marker, correlates with tumor progression in a variety of human cancers. However, the role of CA-IX in hepatocellular carcinomas (HCCs) remains largely unknown. We examined the expression of 277 unifocal, resectable, primary HCC tumors using immunohistochemistry. The CA-IX protein was expressed in 110 of the 227 (48.5%) HCC tumors. The expression of CA-IX correlated with younger age (P = 0.0446), female sex (P = 0.0049), high serum α-fetoprotein levels (P<1x10^-6^), larger tumor size (P = 0.0031), high tumor grade P<1x10^-6^) and high tumor stage (P = 1.5x10^-6^). Patients with HCC tumors that expressed CA-IX were more likely to have lower 5-year disease-free survival (DFS; P = 0.0001) and 5-year overall survival (OS; P<1x10^-6^). The multivariate analysis indicated that CA-IX expression was an independent predictor for high tumor stage (P = 0.0047) and DFS (P = 0.0456), and a borderline predictor for OS (P = 0.0762). Furthermore, CA-IX expression predicted poor DFS and OS in patients with high tumor stage (P = 0.0004 and P<1x10^-6^, respectively). Interestingly, CA-IX expression might contribute to the worse prognosis of female patients with advanced HCCs. Our study indicates the expression of the CA-IX protein is a crucial predictor of poor prognosis in resectable HCC, and it is also an unfavorable prognostic predictor in HCC patients with high tumor stage.

## Introduction

Hepatocellular carcinoma (HCC) is one of the most common cancers worldwide, particularly in Taiwan, southern China, Southeast Asia, and sub-Saharan Africa, and the incidence of HCC is increasing in Western countries [1]. The major risk factors for HCC are hepatitis B and C, liver cirrhosis, and exposure to environmental carcinogens such as aflatoxin [2]. Although surgical resection and various methods of tumor ablation methods can be curative or prolong survival, the outcome for HCC patients remains poor. This is particularly true in patients with advanced- stage HCC because the tumor has often spread throughout the liver via the intrahepatic portal venous system, and a considerable number of HCC patients develop postoperative tumor recurrence [3]. Therefore, the identification of molecular markers that correlate with tumor progression and poor prognosis is crucial to establishing effective treatment plans for HCC patients.

Hypoxia and vascular insufficiency are frequently observed in many types of human cancers. Hypoxia plays critical roles in tumor progression by enhancing epithelial-mesenchymal transition, inducing newly formed vessels that become the main path for tumor metastasis [4,5]. In addition, hypoxia also contributes to the radio- and chemo-resistance of cancer cells [6]. In the molecular level, hypoxia induces the activation of tyrosine kinases such as Src, and the HER2/neu, IGF, and EGF receptors and stimulates the PI3K-AKT-FRAP signal transduction pathway, which leads to increase of hypoxia-induced-factor-1 (HIF-1), and thereafter enhances the transcription of target genes, such as VEGF, IGF-2, and glucose transporters; this results in increased angiogenesis, cell growth, and metabolic processes [7,8]. Moreover, HIF-1 protein binds to hypoxia-responsive element (HRE) and activates the transcription of target genes, such as GLUT1, MCT4, NHE1, VEGFA, PDGF, and carbonic anhydrase IX (CA-IX). These genes further regulate cell growth, microenvironment pH values, angiogenesis, and glucose metabolism, resulting in promotion of tumor progression [7,8].

The CA-IX protein, a direct transcriptional target of HIF-1 and one of the most prominent intrinsic markers of tumor hypoxia [9,10], has been determined to participate in numerous basic physiological functions and cancer processes [11], and can serve as a surrogate marker for the transcriptional activity of HIF-1 in solid tumors [12]. CA-IX facilitates conversion of carbon dioxide to bicarbonate ions and protons, and plays an important role in PH regulation of extracellular microenvironment, which is critical for survival of cancer cells in hypoxia and acidosis [13]. In addition, CA-IX plays an important role in cell migration [14], and its expression has been reported to be a prognostic marker for several types of cancer, including non-small cell lung cancer [15], breast cancer [16], head and neck tumors [17], bladder cancer [18], brain cancer [19], cervical cancer [20], esophageal and gastric cancer [21], rectal cancer [22], soft tissue sarcoma [23], and gallbladder cancer [24]. However, the clinical and pathological significance of CA-IX expression in human HCC remains unclear. The aims of our study were to elucidate the role of CA-IX in vascular invasion, tumor recurrence, and HCC progression, to evaluate CA-IX as a predictive biomarker for survival in HCC patients with high tumor stage.

## Materials and Methods

### Tissue Samples

A total of 227 unifocal, primary HCC tumors surgically resected from patients at National Taiwan University Hospital (NTUH) from July 1988 to September 1996 were used on this study retrospectively. All resected tumors underwent detailed pathological assessment, and all patients received regular follow-up examinations, as described previously [25,26]. Our study was approved by the Ethics Committee of NTUH (approval no. 201309093RIND), and all study procedures were conducted therein. All study participants provided written informed consent, which was approved by the Ethics Committee of NTUH. One teenaged male was included in our study, for whom written, informed consent was obtained from his parents. There were no other minor participants in our study. The anonymity of all patients was maintained, and all specimens were analyzed in a blinded manner. The HCC patients included 177 males and 50 females, with a mean age of 57.2 years (range, 14–88 years). Serum hepatitis B surface antigen (HBsAg) was positive in 145 (64%) patients, and hepatitis C antibody (anti-HCVs) was positive in 75 (33%) patients, 15 of which were positive for both. All patients exhibited adequate liver function reserve at the time that they received curative liver resection, and their records contained complete clinical, histopathological, and follow-up data. No patients had distant metastasis, nor had they received anticancer treatments before undergoing surgery, such as transhepatic arterial chemoembolization, percutaneous ethanol injection, radiofrequency ablation, or chemotherapy.

### Histology and Tumor Staging

Surgically resected specimens were formalin fixed and paraffin embedded. Histological sections were cut at 5-μm-thickness and stained with hematoxylin-eosin. All specimens were reviewed by the same investigator (YMJ) to determine tumor grade and stage. The tumor grading was based on the criteria proposed by Edmondson and Steiner [27]. The tumors were staged according to the American Joint Committee on Cancer (AJCC) system [28] as stages I (99 cases), II (71 cases), and III (57 cases). Because the aim of our study was to evaluate the prognostic value of resectable HCCs, patients classified with stages IVA and IVB were excluded. The margins of the surgical specimens were inked and checked microscopically. Only completely resected specimens were included in our study.

### Immunohistochemical Analysis of CA-IX Protein Expression.

The CA-IX protein was detected in the formalin-fixed, paraffin-embedded sections of HCC and liver tissue using a labeled streptavidin-biotin method after antigen retrieval, as previous studies [29]. The tissue sections were dewaxed and rehydrated. The antigen was retrieved by incubating the slides in 0.01 M citrate buffer (pH 6.0) at 100°C for 15 min. After blocking with 5% fetal bovine serum (FBS), the slides were incubated in a 1:100 dilution of a mouse anti-CA-IX antibody (GTX70020, GeneTex, San Antonio, TX, USA) at 4°C for 16 hours. After washed 3 times with PBS for 5 minute, the slides were incubated with a polymer-HRP reagent (BioGenex, San Ramon, CA, USA) for 30 minutes. The peroxidase activity was visualized using a diamino-benzidine tetrahydroxychloride solution (BioGenex, San Ramon, CA, USA), and the slides were counterstained with hematoxylin. We replaced the primary antibody with 5% FBS as a negative control. In addition, the hepatocytes and bile ducts of the uninfected liver tissues from the surgically resected hepatic hemangiomas were used as negative and positive controls, respectively. One pathologist who was blinded to the patients’ outcomes calculated the percentage of positive cells based on 5 independent microscopic fields (× 400 magnifications) for each slide to ensure the representativeness and homogeneity of all specimens. All tumor cells within each microscopic field were counted, and the positive rate of CA-IX was calculated. For data presentation, the proportion of the tumor cells that was positive for CA-IX immunostaining were categorized as diffuse CA-IX expression (> 50%), focal or heterogeneous CA-IX expression (11–50%), or CA-IX expression in a small proportion of the tumor cells (1–10%). In the nontumorous liver, CA-IX protein was detected only in very few isolated liver cells. Hence, HCC with less than 1% of tumor cells showing immunostaining for CA-IX was regarded as negative.

### Follow-up Observation Examination

All patients had been followed up for more than 5 years or until death, whichever occurred earlier. Among the 227 study patients, 93 (41%) survived longer than 5 years. Following surgery, all patients received laboratory examinations, including assessment of serum α-fetoprotein (AFP) level, at 1- to 6-month intervals, and liver ultrasonography of the liver at 3- to 12-month intervals. Computed tomography (CT) and/or magnetic resonance imaging (MRI) were used to confirm and differentiate intrahepatic recurrence and/or distal metastasis in the patients with clinical signs of recurrence.

Depending on the tumor site, the tumor size, the number of tumors, the level of liver function, and the patient’s condition, tumor recurrence was treated by a second resection, percutaneous ethanol injection, transhepatic arterial chemoembolization, radiofrequency ablation, or chemotherapy. All patients had an equal opportunity to access all the therapeutic modalities supported by the Bureau of National Health Insurance, Taiwan.

### Statistical Analysis

The data analyses were performed using the Epi Info computer software, version 7.1.0.6 (Centers for Disease Control and Prevention, Atlanta, GA, USA). A univariate analysis was used to examine whether the immunohistochemical marker correlated with the clinical and pathological parameters using the χ^2^ and Fisher’s exact tests. The cumulative survival rates after tumor resection were calculated using the Kaplan-Meier method, and the differences in the survival curves were analyzed using the log rank test. A multivariate analysis of all the parameters that were found to be significantly correlated in univariate analysis was performed using logistic regression model for tumor stage, and Cox’s proportional-hazard regression model for disease-free survival and overall survival. A two-tailed *P* value of less than 0.05 was considered to indicate a statistically significant relationship.

## Results

### CA-IX Protein Expression and Distribution in the HCC and Nontumorous Liver Cells

**Fig 1:**
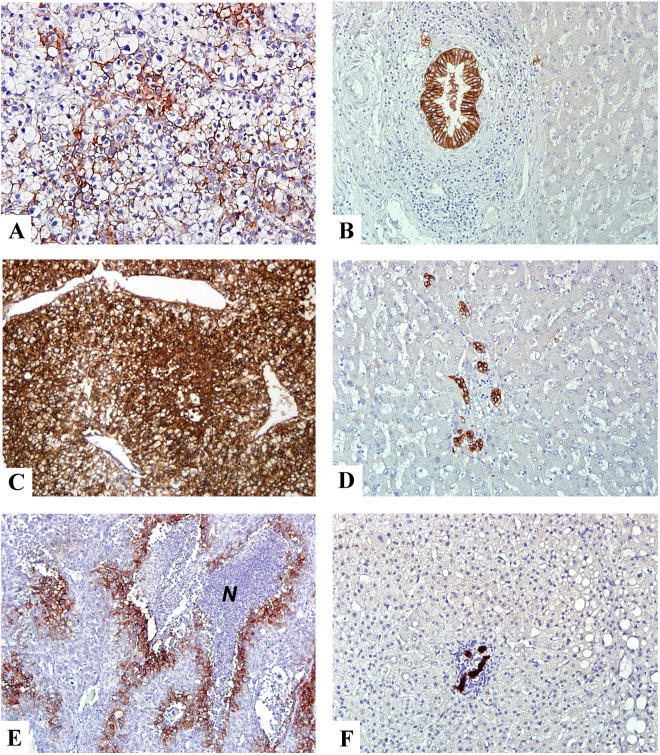
Expression of CA-IX protein in non-cancerous liver parenchyma and HCC. (A and C) Immunohistochemical staining showed heterogeneous and diffuse membranous/cytoplasmic expression of CA-IX in HCC. (E) In the region of tumor necrosis, the viable tumor cells surrounding the necrotic area (*N*) exhibited strong CA-IX expression. (B, D, F) B, D, and F were the nontumorous counterpart of A, C, and E, respectively. They exhibited strong staining in the bile duct epithelial cells in the portal area but not in the hepatocytes or the mesenchymal cells. A-F x200 (original magnification).

The immunohistochemical staining was used to screen 277 HCCs to determine the frequency of CA-IX expression and the clinical and pathological significance of CA-IX expression in HCC. The CA-IX staining was predominantly membranous, with areas of concurrent cytoplasmic and nuclear staining. In the normal or nontumorous liver parenchyma, CA-IX protein was detected only in the biliary epithelial cells but not detected or detected only in very few isolated liver cells (Fig. 1A), which were defined as the negative group (117 cases, 51.5%). CA-IX protein was expressed in a small number of tumor cells in 43 cases (18.9%), focal to heterogeneous in 11–50% tumor cells in 40 cases (17.6%; Fig. 1B), and diffuse in more than 50% tumor cells in 27 cases (11.9%; Fig. 1C), which were regarded as positive group. Moreover, in the positive group, the CA-IX protein expression was predominantly at the periphery of necrotic tumor tissue, which was consistent with the expression pattern of hypoxia markers (Fig. 1D).

### Clinical and Pathological Significance of CA-IX Expression in HCC

To elucidate the significance of CA-IX expression in HCC, we examined possible correlations between CA-IX protein expression and major clinical and pathological features of HCC. Our preliminary analyses showed no statistically significant differences in patient survival and the association with clinical and pathological parameters among patient groups with different degrees of CA-IX expression. Therefore, we dichotomized the expression by negative (<1%) and positive (≥ 1%) only in the following analyses. As presented in Table 1, positive CA-IX expression in the HCCs exhibited a significant association with younger age (≤56 years; *P* = 0.0446), tended to occur in female patients (*P* = 0.0049) and correlated with high serum AFP levels (>200 ng/mL; *P*<1x10^-6^). However, it did not correlate with other clinical parameters, such as serum HBsAg status, and anti-HCV status. Histologically, CA-IX expression exhibited a significant correlation with larger tumor size (>3 cm; odds ratio (OR) = 2.46; 95% confidence interval (CI) = 1.29–4.73; *P* = 0.0031) and high tumor grade (grade III-IV; OR = 5.03; 95% CI = 2.63–9.71; *P* = 0.0005), but not liver cirrhosis. Importantly, high-stage (stage II-III) HCCs, which had vascular invasion and various degree of intrahepatic metastasis, had significant CA-IX expression as compared with low-stage HCCs (stage I; OR = 3.83; 95% CI = 2.11–6.98; *P* = 1.5 x 10^-6^).

**Table 1:** Univariate analysis of CA-IX protein expression with various clinical and pathological features in 227 patients with surgically resectable primary hepatocellular carcinoma.

	CA-IX protein expression			
Variables	Yes n (%)	No n (%)	Odds Ratio (range)	*p* value
Age				
>56	57 (52)	76 (65)	1.72	0.0446
≤56	53 (48)	41 (35)	(0.98–3.04)	
Gender				
Male	77 (70)	100 (85)	2.52	0.0049
Female	33 (30)	17 (15)	(1.25–5.13)	
HBsAg				
Negative	34 (31)	48 (41)	1.55	0.1128
Positive	76 (69)	69 (59)	(0.87–2.79)	
Anti-HCV				
Negative	77 (70)	75 (64)	1.31	0.3451
Positive	33 (30)	42 (36)	(0.72–2.37)	
Cirrhosis				
No	71 (65)	71 (61)	1.18	0.5480
Yes	39 (35)	46 (39)	(0.66–2.10)	
μ-fetoprotein (ng/ml)				
≤ 200	48 (44)	91 (78)	4.52	<1x10^-6^
>200	62 (56)	26 (22)	(2.45–8.40)	
Tumor size (cm)				
≤3	21 (19)	43 (37)	2.46	0.0031
>3	89 (81)	74 (63)	(1.29–4.73)	
Tumor grade				
I-II	54 (49)	97 (83)	5.03	<1x10^-6^
III~IV	56 (51)	20 (17)	(2.63–9.71)	
Tumor stage				
I	30 (27)	69 (59)	3.83	1.5x10^-6^
II-III	80 (73)	48 (41)	(2.11–6.98)	

### CA-IX Expression Predicts Tumor Recurrence and Poor Prognosis

**Fig 2:**
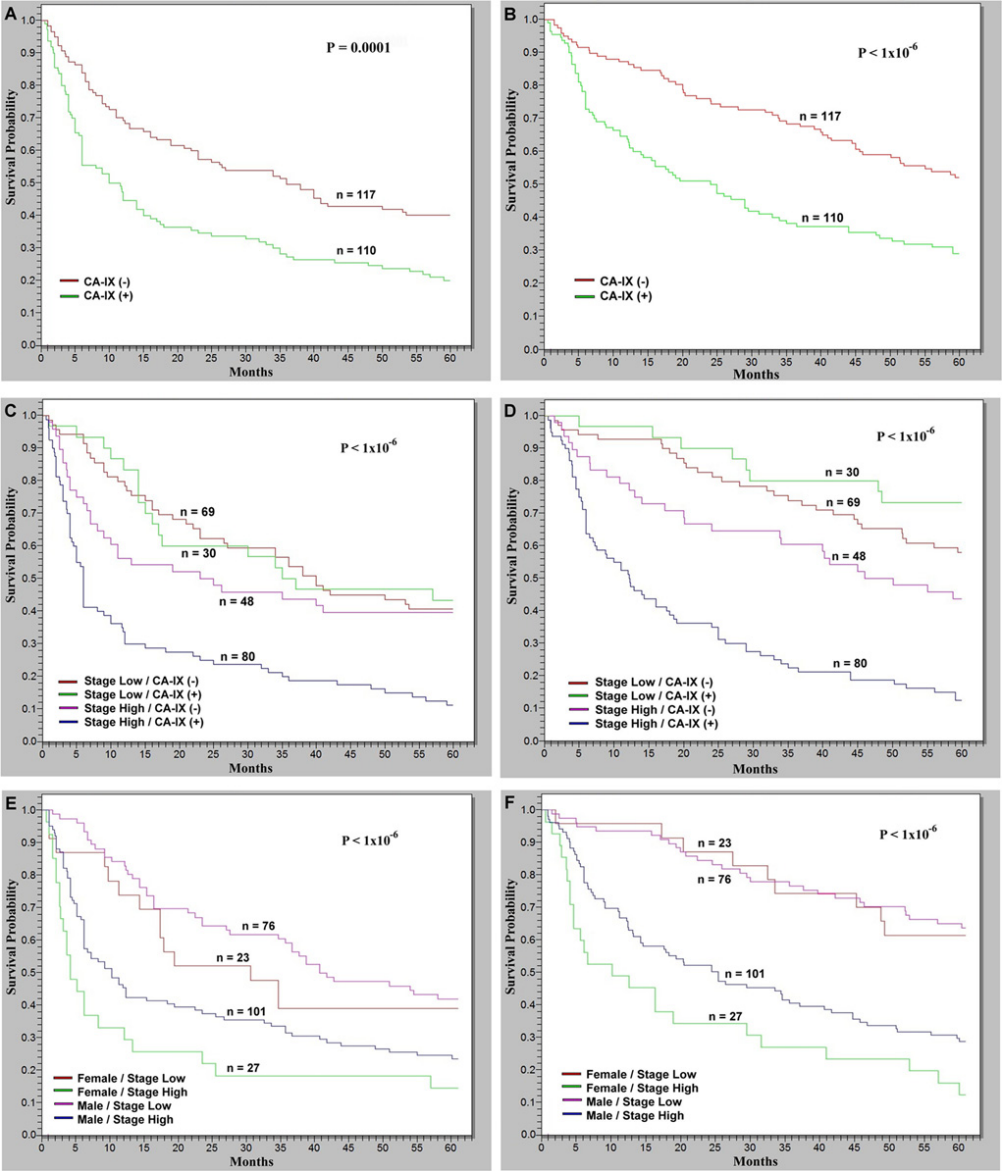
Kaplan–Meier analyses of 5-year disease free survival (DFS) and 5-year overall survival (OS) in 227 patients with HCC. (A and B) The HCCs with CA-IX protein expression correlated with significantly lower DFS and OS than the HCCs without CA-IX protein expression (*P* = 0.0001 and *P*<1x10^-6^, respectively). (C and D) The combinatorial analyses revealed that CA-IX-positive high-stage HCCs had the lowest DFS (*P*<1 x10^-6^) and OF (*P*<1x10^-6^), which were lower than CA-IX negative high-stage HCCs (*P* = 0.0004 and *P*<1x10^-6^, respectively). However, there were no differences concerning both DFS and OS in low-stage HCCs regardless presence or absence of CA-IX expression. (E and F) The female patients presenting with high-stage HCCs exhibited the lowest DFS (*P*<1 x 10^-6^) and OS (*P*<1x10^-6^), which were lower than male patients presenting with high-stage HCCs (*P* = 0.0214 and *P* = 0.0188, respectively). However, there were no differences concerning DFS and OS in male and female patients with low-stage HCCs. (+): The presence of CA-IX protein expression. (-): The absence of CA-IX expression.

Tumor recurrence plays a crucial role in HCC patients with poor prognosis after hepatectomy [26,30]. In our series, tumor recurrence within 5 years occurred in 158 of 227 patients (69.9%). The tumor recurrent rate was approximately 1.34 times higher in the patients with CA-IX-positive HCCs that those with CA-IX-negative HCCs (80.0% *vs*. 59.8%; *P* = 0.0010). The Kaplan-Meier survival analysis indicated that patients presenting with CA-IX-positive HCCs exhibited lower 5-year disease-free survival rate (DFS; *P* = 0.0001; Fig. 2A) and lower 5-year overall survival rate (OS; *P*<1x10^-6^; Fig. 2B) than the patients presenting with CA-IX-negative HCCs.

To identify independent factors for predicting patient survival, we put all the clinical and pathological parameters that exhibited significant correlation with high tumor stage (including age, HBsAg status, Anti-HCV status, liver cirrhosis, serum AFP levels, tumor size, and tumor grade) and significant correlation with patient survival (including age, serum AFP levels, tumor size, tumor grade, and tumor stage) in univariate analysis, and CA-IX expression in multivariate analyses. As presented in Table 2, the results indicated that larger tumor size was an independent predictor for high tumor stage (*P* = 0.0011). In addition, high serum AFP levels, larger tumor size and high tumor stage were independent prognostic factors for DFS and OS (all *P*<0.05). Notably, CA-IX expression was an independent predictor for high tumor stage and DFS (*P* = 0.0047 and *p* = 0.0456, respectively), and was a borderline significant predictor for OS (*P* = 0.0762).

**Table 2:** Multivariate analyses of the risk factors associated with high stage tumor, disease-free survival and overall survival of patients with surgically resectable primary hepatocellular carcinoma.

Covariate	Coefficient	S.E.	Z-Statistic	O.R/H.R. (95% C.I.)	*P-* value
**Stage II-III** ^**†**^					
Age (H/L)	0.4040	0.3292	1.2269	1.4977 (0.7856–2.8556)	0.2199
HBV (P/N)	0.1470	0.4178	0.3519	1.1584 (0.5108–2.6270)	0.7249
HCV (P/N)	-0.1939	0.4271	-0.4541	0.8237 (0.3567–1.9024)	0.3497
Cirrhosis (P/N)	0.5853	0.3429	1.7070	1.7956 (0.9169–3.5163)	0.0878
AFP (L/H)	-0.3731	0.3437	-1.0854	0.6886 (0.3511–1.3507	0.2777
Size (S/L)	-1.2068	0.3689	-3.2717	0.2991 (0.1452–0.6164)	0.0011
Grade (L/H)	-0.4763	0.3533	-1.3482	0.6211 (0.3107–1.2413)	0.1776
CA-IX (P/N)	0.9435	0.3334	2.8302	2.5689 (1.3366–4.9375)	0.0047
**Disease-Free Survival Time** ^**‡**^					
AFP (L/H)	-0.3939	0.1779	-2.2149	0.6744 (0.4759–0.9557)	0.0268
Size (S/L)	-0.5155	0.2019	-2.5533	0.5972 (0.4021–0.8871)	0.0107
Grade (L/H)	0.1734	0.1807	0.9600	1.1894 (0.8347–1.6948)	0.3371
CA-IX (P/N)	0.3660	0.1831	1.9994	1.4420 (1.0072–2.0643)	0.0456
Stage (L/H)	-0.4894	0.1765	-2.7735	0.6130 (0.4338–8663)	0.0055
**Overall Survival Time** ^**‡**^					
AFP (L/H)	-0.4782	0.1895	-2.5230	0.6199 (0.4276–0.8988)	0.0116
Size (S/L)	-0.6824	0.2377	-2.8713	0.5054 (0.3172–0.8053)	0.0041
Grade (L/H)	0.0359	0.1917	0.1870	1.0365 (0.7119–1.5092)	0.8516
CA-IX (P/N)	0.3530	0.1991	1.7729	1.4233 (0.9634–2.1027)	0.0762
Stage (L/H)	-0.9031	0.2049	-0.44070	0.4053 (0.2713–0.6057)	<1x10^-6^

Abbreviations: S.E., Standard error; O.R., Odds ratio; H.R., Hazard ratio; C.I., Confidence interval; AFP, α-fetoprotein; L, Low or Large; H, High; S, Small; P, presence; N, absences.

^†^ Logistic regression model

^‡^ Cox’s proportional hazards model

### CA-IX Expression Predicts Poor Prognosis in Patients with High-Stage Tumors

Although tumor stage is the most crucial factor associated with a poor prognosis, there is heterogeneity in patient prognosis within the same stage group. Therefore, we further analyzed the prognostic role of CA-IX expression in patients with high-stage and low-stage tumors respectively. The combinatorial analysis showed that CA-IX-positive high-stage HCCs had the lowest DFS (Fig. 2C; *P*<1 x10^-6^) and OF (Fig. 2D; *P*<1x10^-6^), significantly lower than CA-IX negative high-stage HCCs (Fig. 2C and 2D; *P* = 0.0004 and *P*<1x10^-6^, respectively). However, there were no differences concerning DFS and OS in patients with low-stage HCCs regardless presence or absence of CA-IX expression.

### CA-IX Expression Contributes to Poor Prognosis in Female Patients with High-Stage Tumors

As presented in Table 1, positive CA-IX expression in the HCCs exhibited a significant correlation with female gender (30% *vs*. 15%; *P* = 0.0049). However, there were no differences in DFS and OS between female and male patients in univariate analysis. Because CA-IX expression can be used as a marker to stratify HCC patients with high-stage tumors, we further analyzed the prognostic role of CA-IX in male and female patients with high-stage HCCs. As shown in Table 3, female patients with high-stage tumors had the highest frequency to have CA-IX expression (*P*<1x10^-6^), significantly higher than male patients with high-stage tumors (81.5% *vs*. 57.4%, *P* = 0.0385), while male patients with low-stage tumors the lowest (25%). The Kaplan-Meier survival analysis indicated that female patients presenting with high-stage HCCs exhibited the lowest DFS and OS (Fig. 2E and 2F; *P*<1 x 10^-6^ and *P*<1x10^-6^, respectively), significantly lower than male patients presenting with high-stage HCCs (Fig. 2E and 2F; *P* = 0.0214 and *P* = 0.0188, respectively). However, there were no differences concerning DFS and OS in male and female patients with low-stage HCCs.

**Table 3:** Analysis of CA-IX protein expression in 227 patients with surgically resectable primary hepatocellular carcinoma stratified by gender and tumor stage

Feature	Female / H	Female / L	Male / H	Male /L	*P* value
**CA-IX expression**					
Yes	22 (81.5%)^a^ ^,^ ^b^	11 (47.8%)^a^	58 (57.4%)^b^	9 (25.0%)	<1x10^-6^
No	5 (18.5%)^c^	12 (52.2%)	43 (42.6%)	57 (75.0%)^c^	

Abbreviations: H, high tumor stage; L, low tumor stage.

a, b, and c designate comparison between the indicated two groups.

*P* values:

^a^0.0275;

^b^0.0385;

^c^< 1x10^-6^

## Discussions

Hypoxia is a major stimulus of angiogenesis, fibrogenesis, and tumor progression [31]. In clinical settings, HIF-1α is considered a target for HCC therapy, and the overexpression of HIF-1α is associated with poor prognosis in HCC patients [32,33]. HIF-1α directly activates TWIST to promote the epithelial-mesenchymal transition and tumor metastasis [4]. Hypoxia induces the excessive accumulation of lactate and protons and deceases extracellular pH, leading to microenvironment that is conductive to the promotion of tumor motility, invasion, and metastasis [5]. A reduction in fatty acid oxidation accompanied by hypoxia may affect the progression of HCCs [34]. Moreover, tumor hypoxia is known to be associated with resistance to chemotherapy and radiotherapy [6]. CA-IX is a downstream target of HIF-1and is a marker for tissue hypoxia. Inhibition of the major kinase pathways regulating HIF-1 resulted in abrogation of CA-IX promoter activity [35]. Kaluz et al. reported that CA-IX is one of the most sensitive endogenous sensor of HIF-1 activity due to the unique structure of CA-IX promoter and therefore CA-IX expression can represent the transcriptional activity of HIF-1 [12].

In this study, we evaluated the clinical and pathological significance of CA-IX expression in HCCs and determined that positive CA-IX expression in the HCCs exhibited a significant correlation with high serum AFP level, which is crucial clinical and histopathological risk factor for poor prognosis [30]. In addition, positive CA-IX expression also exhibited a significant correlation with high tumor grade. This finding is similar to the correlation of CA-IX expression with high tumor grade in clear cell renal cell carcinoma [36] and basal-like breast cancer [37]. Furthermore, immunohistochemical staining showed that CA-IX protein was predominantly expressed at the periphery of necrotic tumor tissue which indicates that tumor cells with CA-IX expression are more resistant to hypoxia and possess a growth advantage. Collectively, these findings indicate that CA-IX is involved in cell proliferation and differentiation, and its expression in HCC facilitates tumor cell growth and contributes to poor differentiation of the tumor cells, and hence high AFP. These findings are also consistent with our previous observations that indicated a correlation between high AFP levels and poorly differentiated HCCs [30].

Studies on CA-IX expression in HCCs are sparse. Luong-Player et al. found that CA-IX expressed focally in only 15% of HCCs and its expression may be useful in differentiating HCC from intrahepatic cholangiocarcinoma [38]. Yu et al. demonstrated that CA-IX expressed in 30.4% of HCCs, but neither the extent nor the intensity of CA-IX immunoreactivity correlate with clinicopathological variables [39]. In our study, we found that CA-IX expressed in 48.5% of HCCs, and its expression significantly correlated with serum AFP levels, tumor size, tumor grade, tumor stage, and patient survival. Although the reasons for these discrepancies are unknown, they might be related to the quality of the antibodies, the details of the staining techniques, and the differences in the study population. In addition, the number of cases in those studies was considerably small (n = 34 and n = 69, respectively). Our study had a much larger cohort, which may make statistical significance much easier to identify. Besides, all the patients reported by Yu et al. had received TACE before surgical resection whereas we chose only patients without previous treatment as our study subjects.

In addition to be a surrogated marker of tissue hypoxia, CA-IX protein was shown to play important roles in tumor progression. Yu et al. demonstrated that the intensity of CA-IX immunoreactivity is inversely related to E-cadherin intensity [39]. They postulated that CA-IX expression may have prognostic implications in HCC patients because downregulation of E-cadherin decreases intercellular adhesion, provides the possibility of epithelial-mesenchymal transition, and results in invasiveness, metastasis and poor progression in HCC. However, they did not demonstrate the prognostic significant of CA-IX expression directly [39]. Fiaschi et al. demonstrated that cancer-associated fibroblasts increase extracellular acidity and result in metabolic reprogram and epithelial-mesenchymal transition, motility, survival and stemness of cancer cells in prostate cancer [40]. In a recent study, CA-IX is found to be essential for the maintenance of cancer stem cells, and targeting carbonic anhydrase IX depletes breast cancer stem cells within the hypoxic niche [41]. CA-IX expression is positively correlated with resistance to chemotherapy in basal-like breast cancer [37], and is also a significant and independent prognostic indicator of overall survival and metastasis-free survival after radiation therapy in cervical cancer [20] and breast cancer [42]. On the contrary, inhibition of CA-IX activity can enhance the effect of tumor irradiation [43]. Even though the major limitation of this study is that we did not prove the mechanical association of CA-IX expression with hepatocarcinogenesis, these findings suggest possible mechanical association and provide us a rational for a new therapeutic strategy by downregulation of CA-IX expression to improve cancer therapy in HCC patients.

Tumor stage is a crucial histopathological factor in determining prognosis [44,45]. In this study, we found that CA-IX expression is an independent predictor for high-stage HCCs. Consistent with the reports regarding various human cancers [15–24], we demonstrated for the first time that CA-IX-positive HCCs were associated with significantly lower DFS and OS than CA-IX-negative HCCs in patients with resectable tumors. These findings also drove us to search for the prognostic factors in patients with high-stage HCCs. We found that patients with CA-IX-positive high-stage HCCs had the worst DFS and OS, significantly worse than patients with CA-IX-negative high-stage HCCs. These findings indicated that CA-IX exerted additional adverse effects on patients’ survival even in those patients with high-stage HCCs. This finding is similar to the report that CA-IX expression is an significant prognostic factor for in patients with advanced head-and-neck cancer [46]. Our results suggested that CA-IX immunostaining can divide HCCs of the same stage into different prognostic groups, and may help clinical management of patients by a more accurate prediction of patient survival, and will help us to develop better patient management strategies.

In our study, positive CA-IX expression in the HCCs exhibited a significant correlation with female patients. This finding is in accordance with the previous report by Eckert et al. that female patients show a significantly higher expression of CA-IX than male patients in oral squamous cell carcinomas [47]. Even though we did not demonstrate favorable DFS and OS in female HCC patients, female gender is a well-known prognostic factor in both treated [48] and untreated [49] HCC patients. Recently, Giannini et al. demonstrated that female gender is an independent prognostic predictor in untreated patients with advanced HCCs [50]. Interestingly, we found that female gender was a significant prognostic predictor for DFS and OS in patients with resectable advanced HCCs. In our study, we found that female patients with high-stage HCCs had the highest frequency of CA-IX expression, even higher than male patients with high-stage HCCs. We believed that, to some extent, positive CA-IX expression might contribute to the worse prognosis of female patients with resectable advanced HCCs. However, further studies were mandatory to clarify the roles of CA-IX expression in hepatocarcinogenesis in the female patients with advanced HCCs.

In conclusion, our study revealed that CA-IX expression is an important molecular predictor for postoperative tumor recurrence, and thus a prognostic indicator of unfavorable outcome, and can be a potential therapeutic target for HCC. In addition, the combinatorial analysis indicated a worse prognosis in patients with high-stage HCCs presenting CA-IX expression. Because CA-IX is an enzyme overexpressed in cancer and specific inhibitors have been developed, further studies targeting CA-IX should be performed to test the possibility of inhibiting CA-IX function for the treatment of HCC patients.
